# Results of an Inpatient Preventive Health Care Program to Improve Quality of Life, Psychosocial Health, and Work Ability in Austria

**DOI:** 10.3389/ijph.2023.1606193

**Published:** 2023-09-15

**Authors:** Bettina Thauerer, Johannes Püspök, Werner Kullich, David Felder, Bibiane Steinecker-Frohnwieser, Martin Skoumal

**Affiliations:** ^1^ Ludwig Boltzmann Institute for Arthritis and Rehabilitation, Saalfelden, Austria; ^2^ Moorheilbad Harbach Health and Rehabilitation Center, Lauterbach, Austria; ^3^ Department of Rehabilitation Research, Austrian Federal Pension Insurance (PVA), Vienna, Austria; ^4^ Austrian Federal Pension Insurance (PVA), Vienna, Austria

**Keywords:** preventive health program, “Gesundheitsvorsorge-Aktiv”, quality of life, psychosocial health, strengthening self-responsibility, occupational participation

## Abstract

**Objective:** The Austrian Federal Pension Insurance (PVA) developed a preventive inpatient health program, “Gesundheitsvorsorge-Aktiv (GVA),” for patients with musculoskeletal disorders. Individualized modular interventions and therapeutical measures (movement optimization, movement motivation, and mental health) are designed to improve occupational participation by influencing lifestyle factors and health-related quality of life. The study aimed to evaluate the new prevention-oriented and more personalized inpatient health program GVA.

**Methods:** Patients underwent a standard inpatient health program, with emphasis on exercise management, exercise motivation, or psychological aspects. Submodule-dependent outcomes were assessed in patients (*n* = 330) at the start, end of treatment, and 6 months thereafter. Quality of Life (EQ-5D-5L), psychosocial aspects of the Patient Health Questionnaire (PHQ-D), and Work Ability Index (WAI) were queried.

**Results:** The results consistently showed positive short and long-term effects. The subjective assessments of current work ability improved while the impairment of work performance was reduced. Positive changes in the psychosocial sphere were observed, alongside improvements in the health-related quality of life. Patients in the exercise optimization module performed better in all respects.

**Conclusion:** In summary, GVA represents a valuable preventive health measure that leads to a holistic increase in well-being and can also ensure the maintenance of the ability to work.

## Introduction

Nowadays, it is particularly important to function in the professional sphere. Time has become more fast-moving and debilitating, partly justified by demographic change leading to a higher average age plus the greater demands of working life also influencing health [1]. Health, meaning functional and performance capacity, is becoming particularly important. The presence of persistent diseases or chronic health problems can have a critical impact on health-related quality of life. In particular, work-related musculoskeletal disorders, such as back pain and back disorders, are responsible for a decline in work ability, making work-related musculoskeletal disorders one of the most costly health problems in modern industry [2]. Therefore, health-related life quality plays an increasing role as an indicator of health in the population. It reflects the physical, mental, social, and environmental components of well-being as well as the functional capacity from the subjective perspective of those affected. People with chronic diseases or health problems have a lower quality of life, especially in terms of general condition, and physical and mental well-being [3]. Obesity, mental illness, and chronic musculoskeletal conditions are an increasing public health burden globally. Chronic back problems are mainly responsible for the increase in the number of years lived with disability (YLD) [4]. In 2019, 1.9 million people in the Austrian population (26%) suffered from back pain [3]. People aged between 40 and 69 years are especially affected [5]. Together with diseases of the respiratory system, musculoskeletal diseases cause approximately 50% of sick leave cases and 42% of all sick leave days in Austria [6]. Preserving healthy years, being able to work, and avoiding the need for long-term care are of paramount importance for each individual, as well as the financial viability of the social insurance system. Regular physical activity serves to satisfy the biologically determined need for movement [7] and can improve health, help manage weight, reduce disease risk, and strengthen bones and muscles. Not many lifestyle changes have such an impact on health as physical activity, which can subsequently reduce costs [8]. A guide paper on this topic has been published [9]. In Austria, physical activity and sports have been recognized as an important component of national public health measures, and “promoting healthy and safe physical activity in everyday life” is one of 10 national health goals [10]. The WHO and the European Commission regularly refer to the high importance of physical activity and call for more attention to be paid to physical activity promotion at a national level. In particular, the change from “physically inactive” to “some physical activity” is an important step [11]. To address these health challenges, the Austrian Federal Pension Insurance (PVA) developed an inpatient, exclusively preventive health promotion program, “Gesundheitsvorsorge-Aktiv (GVA),” which has been implemented and funded throughout Austria since 2018. The GVA program is offered to employed and retired individuals with musculoskeletal problems not requiring specialized rehabilitation services. The goal of the GVA program is to optimize patients’ ability to function. “Gesundheitsvorsorge-Aktiv” is a high-quality preventive health measure for musculoskeletal disorders that offers a 22 days inpatient stay and enables a more targeted approach to patients’ individual problems through a broad range of services based on the WHO’s International Classification of Functioning, Disability and Health (ICF) biopsychosocial model [12, 13]. The ICF represents a paradigm shift from a purely biomedical perspective to a perspective of a person’s lived experience of health in the context of a health condition. It provides a universally shared conceptual basis for various fields in health sciences and practice, is a frame of reference for operationalizing health, and is important for standardized reporting of health outcomes, for quality management of clinics and services, and for evidence-based health policies [14–16]. It can be seen that the ICF has been used worldwide as a global framework for describing functioning and disability since its adoption in 2001 [17]. The primary goal is to promote and develop health literacy and make people aware that they can actively contribute to their own health. By consolidating and maintaining health and strengthening self-care, sustainable sociomedical effects can be achieved with regard to maintaining the ability to work and avoiding the need for long-term care. Through separate modules on exercise optimization, exercise motivation, and mental health, the focus of the GVA is on improving lifestyle factors in these areas, in addition to treating the main disease in the musculoskeletal system. Active therapies and adapted exercise form the medical basis for strengthening personal responsibility, integrating exercises and practiced sporting activity into everyday life, and implementing them on a permanent basis. In addition, nutrition in everyday life and workshops on the topics of (occupational) everyday life and healthy/self-determined life are included. This is intended to strengthen the patients' personal responsibility for maintaining their health, coping with stress and frustration, and dealing with the demands of everyday (work) life [18]. The preventive measures of the GVA serve to consolidate and improve health, thus maintaining an individual’s capacity to participate in private and professional life to the greatest extent, while patients are encouraged to actively contribute to and take personal responsibility for their own health.

This study aimed to evaluate the GVA preventive program with a detailed analysis of the modules included in the inpatient program and thus characterize a success depending on the allocation to the modules. For this, the following assessment tools were used at the start, end of the health care program, and 6 months after the program ended: Work Ability Index (WAI), psychosocial aspects of the Patient Health Questionnaire (PHQ-D), and EQ-5D-5L for quality of life.

## Methods

### GVA Program

The modular 3 weeks inpatient program is preventative, is not a post-treatment after illness/injury/surgery, and is offered to all patients with musculoskeletal disorders. Each patient receives at least 1,400 min of therapy over the 3 weeks of the program. Therefore, patients are primarily classified based on their own assessment. Units of exercise-therapy, strength-, endurance-, or relaxation-training form the basic module, which is precisely defined in a service profile. During the intake interview by the physician, the patient can decide for the first time, together with the support of an expert (physician) which of the three modules can best help in the current situation. The evaluation of the individual modules and their comparison was particularly valuable to learn how well this personalized assignment to one of the three modules and their implementation worked.

The exercise optimization module (module 1) is suitable for patients who already exercise regularly. Under the guidance of qualified therapists, exercise plans, their sequences, and routines are optimized to avoid harmful long-term effects on the musculoskeletal system.

The exercise motivation module (module 2) is suitable for people who report little pleasure in exercising and rarely engage in physical activity in everyday life. The aim of this module is to promote enjoyment and motivation for regular physical activity. Both modules are characterized by an increased proportion of exercise-promoting therapies for an average of 2–3 h per day. Active sessions consist of physical activities such as gymnastics and individual physiotherapy, while exercise therapy focuses on underwater, ergometer, Nordic walking, strength, balance, relaxation, and movement training. Passive treatments include massages, thermotherapy, electrotherapy, and ultrasound.

The mental health module (module 3) is aimed at patients who experience psychological stress in everyday life, in their social environment or at work, and focuses on stress prevention, learning relaxation techniques, and distancing mechanisms, as well as strengthening self-protection mechanisms. Patients are accompanied by psychologists, which allows for more targeted and individualized treatment of problems.

The face-to-face nature of the program ensures constant supervision by doctors, psychologists, and physiotherapists, including individual therapy sessions, and is provided during the 3 weeks of the program. This is supplemented by workshops on the topics of healthy and balanced nutrition, occupational stress, and staying active and independent with age. In addition, nutrition in everyday life and workshops on the topics of (professional) everyday life and healthy/self-determined life are included. This is intended to strengthen the patients’ personal responsibility for maintaining their health, their ability to cope with stress and frustration, and to deal with the demands of everyday (work) life. By consolidating and maintaining health, as well as strengthening self-care, sustainable sociomedical effects can be achieved in terms of maintaining the ability to work and avoiding the need for long-term care.

In the time between discharge and follow-up, no guided exercises were offered and patients were on their own and it was up to them how far they integrated and implemented what they had learned into everyday life.

### Study Design and Assessment Tools

In this monocentric, prospective pre-post evaluation, employed and retired individuals were recruited at the health and rehabilitation center (Moorheilbad Harbach, Lower Austria, Austria) from 1 January 2020 up to 29 September 2020 after signing a written informed consent. Patients could refuse to participate in the study at any time without giving a reason, thus withholding their consent to the processing of routinely collected data. The inpatient program lasted 3 weeks (22 days) for each patient. The 6 month follow-up was announced by telephone and the GVA questionnaires were sent by mail. The last contact was on 29 March 2021. Patients included in the study participated in a preventive program specifically established in the Austrian rehabilitation landscape. The majority of the surveys were routinely administered via the hospital information system management in accordance with the Declaration of Helsinki in the current valid version. Upon arrival, patients were fully informed about the extent and purpose of the study, agreed to complete questionnaires during the course of their stay, and signed a privacy statement. Patients were not subjected to any intervention specifically set up for the study, and no patient was deprived of therapy, which means that from an ethical point of view, none of the patients faced disadvantages. An ethics review was not required according to the legal regulations for research in Austria.

Three time points were chosen at which the patients were interviwed with the paper pencil GVA questionnaire: T_0_ (admission), T_1_ (discharge), and T_2_ (6 month follow-up). Sociodemographic and health-related behavior, quality of life (EQ-5D-5L index), general health (EQ-5D-5L VAS), psychosocial components (based on PHQ-D) and subjective ability to work (WAI), among others, were collected.

#### Assessment Tool for Quality of Life and General Health

The EQ-5D-5L, a generic measurement instrument eliciting health status through a standardized preference-based procedure [19], was applied. Health status, described using five dimensions (mobility, self-care, usual activities, pain/discomfort, and anxiety/depression), each with five response levels (no problems, slight problems, moderate problems, severe problems, and extreme problems), and current health status, using a visual analog scale (VAS) in the standard layout of a 20 cm vertical scale with a score range of 0–100, were assessed. Health status can be expressed as a 5-digit code by converting the answers [20] or an unidimensional index value ranging from less than 0 (where 0 is the value of health state equivalent to dead and negative values represent worse than dead) to 1 (the value of full health), which reflects how good or bad the health status is in relation to the population of a country. The EQ-5D-index can be used in Quality-Adjusted-Life-Years (QALY) evaluations, as well as a stand-alone index in health economic evaluations [21].

#### Analysis of Psychosocial Components

The PHQ-D, suitable for initial diagnosis and for the assessment of the course of mental disorders, was used as a screening instrument. Relevant items were implemented and an evaluation was carried out according to the severity of depression, symptom severity/somatization, and psychosocial stress factors [22]. The scale in the depression component (0–27) indicates the severity of depression: no (0), minimal (1–4), mild (5–9), moderate (10–14), and severe depressive symptoms (15–27). Somatic symptoms could be revealed by the somatoform component. A scale score of 0–30 was summed to indicate the severity of the most common somatic symptoms: minimal (0–4), mild (5–9), moderately pronounced (10–14), and severe somatic somatization (15–30) [23]. Answers to the stress component resulted in a scale sum value of 0–20, which gives information about the severity of the stress factors: minimally pronounced psychosocial stressors (0–4), mildly pronounced psychosocial stressors (5–9), moderately pronounced psychosocial stressors (10–14), and severe psychosocial stressors (15–20).

#### Analysis of Work Ability

Work ability defines the ability to perform an activity at work depending on a person’s physical and mental resources and is operationalized using the Work Ability Index (WAI). The WAI, with increasing importance in recent years, is an internationally freely available self-assessment instrument in which work ability is conceived as a multidimensional construct (seven dimensions, each captured by one or more questions) [24–26]. Values were assigned according to the ticked answers and an evaluation was done by summing up all the points achieved in each dimension: the highest score was 49 (maximum work ability) and the lowest score was 7 (minimum work ability). To perform a calculation of the index, all questions had to be answered. Half points in the total score were rounded up to the nearest whole value.

### Statistical Analysis

All statistical analyses were performed with the statistical program GraphPad Prism version 9.5.0. Continuous variables are expressed as mean (standard error of mean, SEM), and categorical data (frequency distribution) as counts (percentage, %). Normal distribution was assessed using the Shapiro-Wilk test. Groups were compared using the non-parametric Kruskal Wallis test, and paired comparisons within groups were done with the Friedmann test. No correction for multiple comparisons was performed and each comparison stands alone, in addition, an uncorrected Dunn´s test was used. The correlation was assessed using Spearman´s rank correlation coefficient following simple linear regression. The *p*-value was considered significant if <0.05. Participants with missing data were excluded.

## Results

### Participants

**FIGURE 1:**
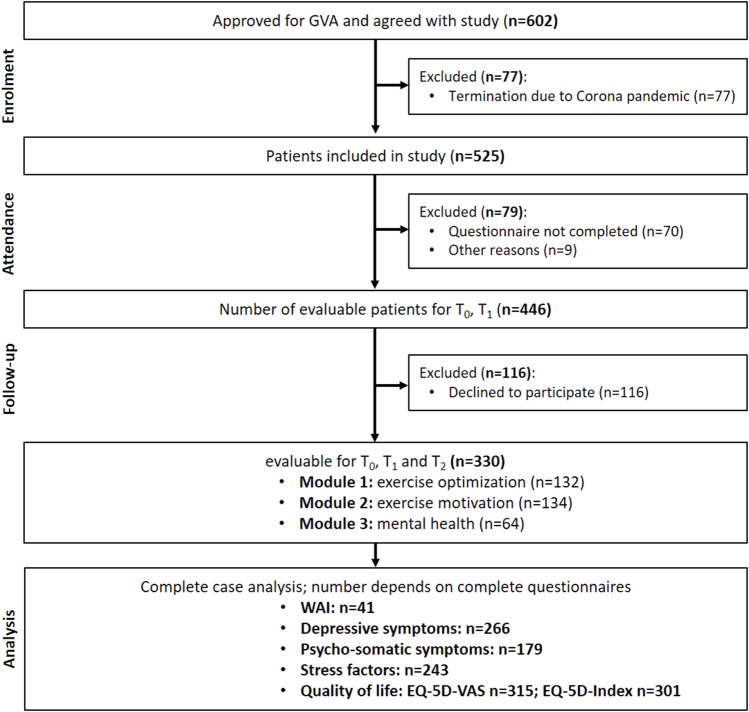
Flow-diagram of participants through the study (GVA, Austria 2020).

All patients who consented to the study received a GVA questionnaire at the beginning of the program T_0_ (*n* = 602). At the end of the measures (T_1_), 446 patients completely filled out the questionnaires. Drop out reasons were corona-related termination, uncompleted questionnaires, and others. Furthermore, 116 people declined to participate in the 6 month follow-up and did not return the questionnaire. Finally, 330 complete datasets were evaluable for all three time points (T_0_, T_1_, and T_2_). Considering this, the dropout rate was 45%. Subgroup analysis was performed based on the physician’s classification into submodules (Figure 1).

### Demographic Data

Subgroups regarding the physician’s classification were formed: exercise optimization (*n* = 132; 40%), exercise motivation (*n* = 134; 41%), and mental health (*n* = 64; 19%) (Figure 1). The average age did not differ between the modules. The sex distribution was relatively balanced in the module for exercise optimization, but there were more men in the group for exercise motivation. The very high proportion of women, approximately 66% (*n* = 42), in the mental health module was striking. The median BMI was below the obesity threshold of 30 kg/m^2^ in all modules and thus within the body mass range specified by the WHO as overweight, but it is worth mentioning that the highest BMI was detected in patients of the exercise motivation module. There were more smokers in the exercise motivation and mental health modules. Significant differences were found in the distribution of occupational status by comparing the exercise optimization module with the exercise motivation or mental health modules (*p* < 0.05) (Table 1).

**TABLE 1:** Demographic characteristics assessed at T_0_ (GVA, Austria 2020).

	Module 1: Exercise optimization	Module 2: Exercise motivation	Module 3: mental health
Number of patients, n	132	134	64
Age (years), mean ± SEM [median]	52.8 ± 0.7 [55.0]	53.8 ± 0.5 [54.5]	52.7 ± 0.8 [54.0]
BMI (kg/m^2^), mean ± SEM [median]	27.0 ± 0.4 [26.4]	30.0 ± 0.5 [29.4]	28.1 ± 0.7 [27.0]
Female, n [%]	68 [51.5]	59 [44.0]	42 [65.6]
Male, n [%]	64 [48.5]	75 [56.0]	22 [34.4]
Smoking, n [%]	14 [10.7]	28 [21.1]	23 [35.9]
Occupation			
employed, n [%]	117 [88.6]	103 [78.0]	48 [75.0]
unemployed, n [%]	7 [5.3]	22 [16.7]	14 [21.9]
Pension, n [%]	8 [6.1]	6 [4.5]	1 [1.6]
Rehab allowance, n [%]	0 [0]	1 [0.8]	1 [1.6]

Abbreviations: BMI—body mass index; n—number of participants; Kruskal Wallis Test (significances compared to exercise-optimization-module): *p < 0.05.

### Sportiness

**FIGURE 2:**
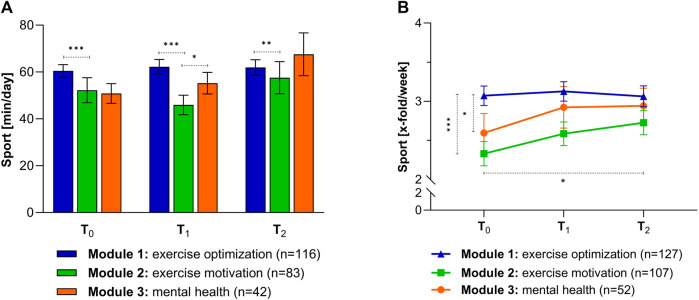
Differences between the individual modules in terms of sports: **(A)** Duration (min/day) at different time points. Values are given as mean ± SEM. Kruskal Wallis test: **p < 0.01 (T_2_: exercise optimization vs. exercise motivation), ***p < 0.001 (T_0_ and T_1_: exercise optimization vs. exercise motivation). **(B)** Differences in frequency (x-fold/week) among surveys. Values are given as mean ± SEM. Kruskal Wallis test: *p < 0.05 (T_0_: exercise optimization vs. mental health), ***p < 0.001 (T_0_: Exercise optimization vs. exercise motivation); Friedmann test: *p < 0.05 (exercise motivation: T_0_ vs. T_2_). (GVA, Austria 2020).

Comparing the three modules, significant differences in duration and frequency at time point T_0_ could be seen (Figure 2A,B). Initial deficits, in terms of sports time per day, were minimized. An increase in physical activity per week (Sport [x-fold/week]) was observed in the exercise motivation and mental health modules, on the other hand, no change was recognized in the optimization module by the GVA program. Participants from the exercise motivation and mental health modules maintained these improvements after 6 months (Figure 2B). No differences were noticeable between the participants of the modules in the 6 month follow-up.

### Psychosocial Components

**FIGURE 3:**
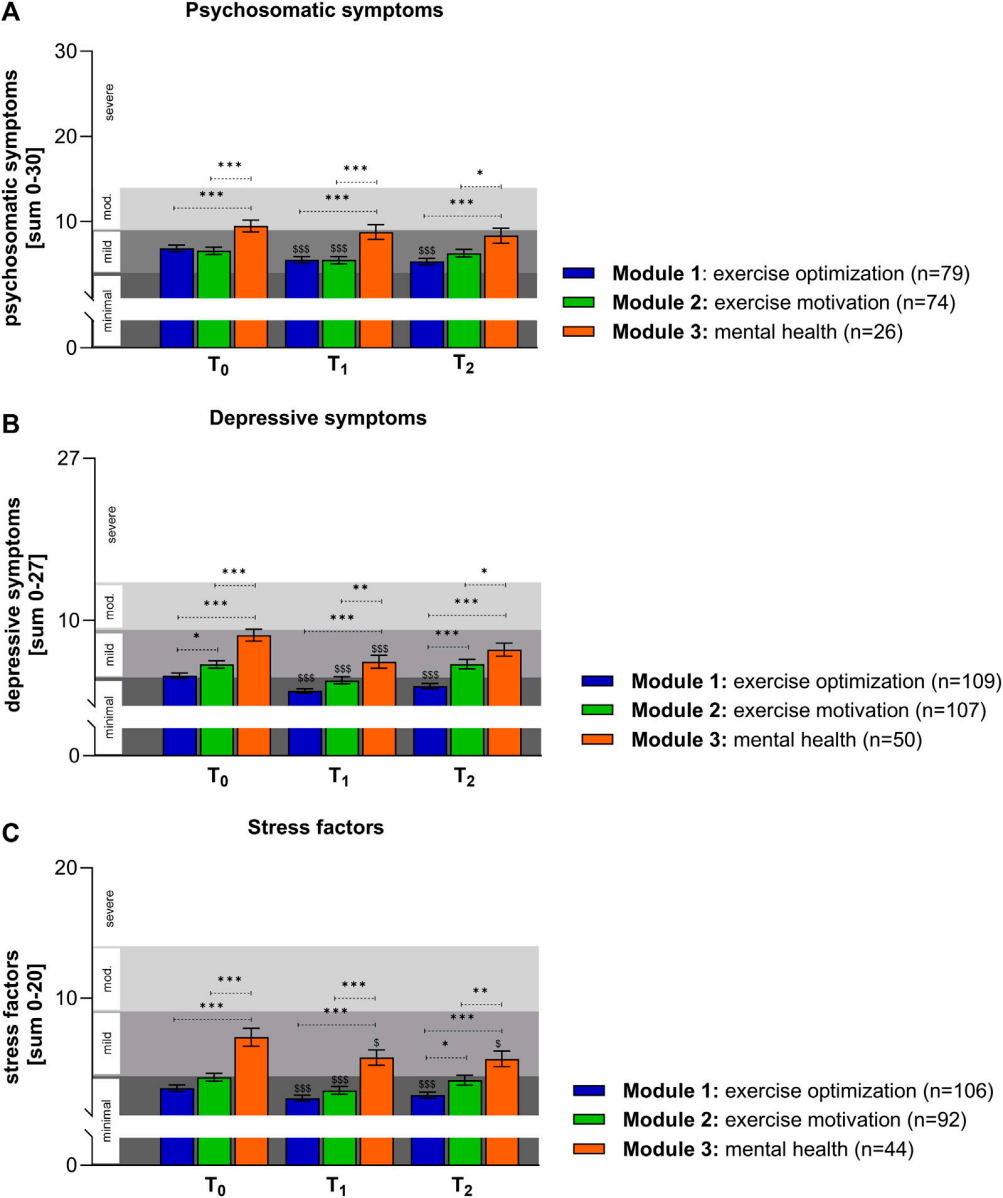
Psychosocial differences between the individual modules: **(A)** Patients of the mental health module showed higher values in psychosomatic symptoms. Time course-dependent reductions for psychosomatic symptoms are shown. Values are given as mean ± SEM. Kruskal Wallis test: *p < 0.05 (T_2_: exercise motivation vs. mental health), ***p < 0.001 (T_0_, T_1_ and T_2_: exercise optimization vs. mental health), ***p < 0.001 (T_0_ and T_1_: exercise motivation vs. mental health). Friedman test: ^$$$^ *p* < 0.001 (exercise optimization: T_0_ vs. T_1_ and T_0_ vs. T_2_), ^$$$^ *p* < 0.001 (exercise motivation: T_0_ vs. T_1_). **(B)** The GVA program is very effective in reducing depressive symptoms in all three offered modules. Values are given as mean ± SEM. Kruskal Wallis test: *p < 0.05 (T_0_: exercise optimization vs. exercise motivation), *p < 0.05 (T_2_: exercise motivation vs. mental health), **p < 0.01 (T_1_: exercise motivation vs. mental health), ***p < 0.001 (T_0_, T_1_ and T_2_: exercise optimization vs. mental health), ***p < 0.001 (T_0_: exercise motivation vs. mental health). Friedman test: ^$$$^ *p* < 0.001 (exercise optimization: T_0_ vs. T_1_ and T_0_ vs. T_2_), ^$$$^ *p* < 0.001 (exercise motivation: T_0_ vs. T_1_), ^$$$^ *p* < 0.001 (mental health: T_0_ vs. T_1_). **(C)** Significant changes in stress factors within the measured time points are shown. Values are given as mean ± SEM. Kruskal Wallis test: *p < 0.05 (T_2_: exercise optimization vs. exercise motivation), **p < 0.01 (T_2_: exercise motivation vs. mental health), ***p < 0.001 (T_0_, T_1_ and T_2_: exercise optimization vs. mental health), ***p < 0.001 (T_0_ and T_1_: exercise motivation vs. mental health). Friedman test: ^$$$^ *p* < 0.001 (exercise optimization: T_0_ vs. T_1_ and T_0_ vs. T_2_), ^$$$^ *p* < 0.001 (exercise motivation: T_0_ vs. T_1_), ^$^ *p* < 0.05 (mental health: T_0_ vs. T_1_ and T_0_ vs. T_2_). (GVA, Austria 2020).

In order to analyze the different situations in psychosocial components between the three offered modules, scales for psychosomatic symptoms, depressive symptoms, and stress factors were formed at the beginning of the GVA measures (T_0_) and at the following time points, T_1_ and T_2_ (Figures 3A–C). Individuals from the exercise optimization and exercise motivation modules had significantly better conditions in terms of psychosomatic symptoms at the beginning, with conditions located in the mild range, than those from the mental health module (Figure 3A). By analyzing the subsequent time points, an improvement was shown by the GVA program. A clear short-term decrease in psycho somatic symptoms of approximately 20% and an additional 3% longer-lasting reduction were seen in participants of the exercise optimization module. A reduction was also evident in the motivation module, but this was hardly present after 6 months. In the mental health module, no significant changes were seen in this component. By looking at the depressive symptoms, patients in the mental health module showed a value in the higher mild range at the beginning (Figure 3B). A significant decrease in depressive symptoms in all modules within the 3 weeks program was observed. The most prominent effect could be seen in the participants of the exercise optimization module with a reduction of up to 38%, followed by the mental health module with 33%, and the exercise motivation module with a 31% decrease. These reductions could be observed also at the 6 month follow-up (T_2_), however, attenuated. People who were initially classified by the doctor in the mental health module showed a nearly 2-fold higher value of stressors at the beginning of the program than people in the other modules (Figure 3C). A minimization of various severe stress factors could be achieved with the measures in the exercise optimization module (25%), exercise motivation module (26%), and the mental health module (22%). The latter positive effect was also visible after 6 months.

For all psychosocial components examined, such as somatoform symptoms, depressive symptoms, and stressors, those who were already athletic and only needed optimization performed best. This group also showed psychosocial improvements up to 6 months following the GVA program.

### Quality of Life

**FIGURE 4:**
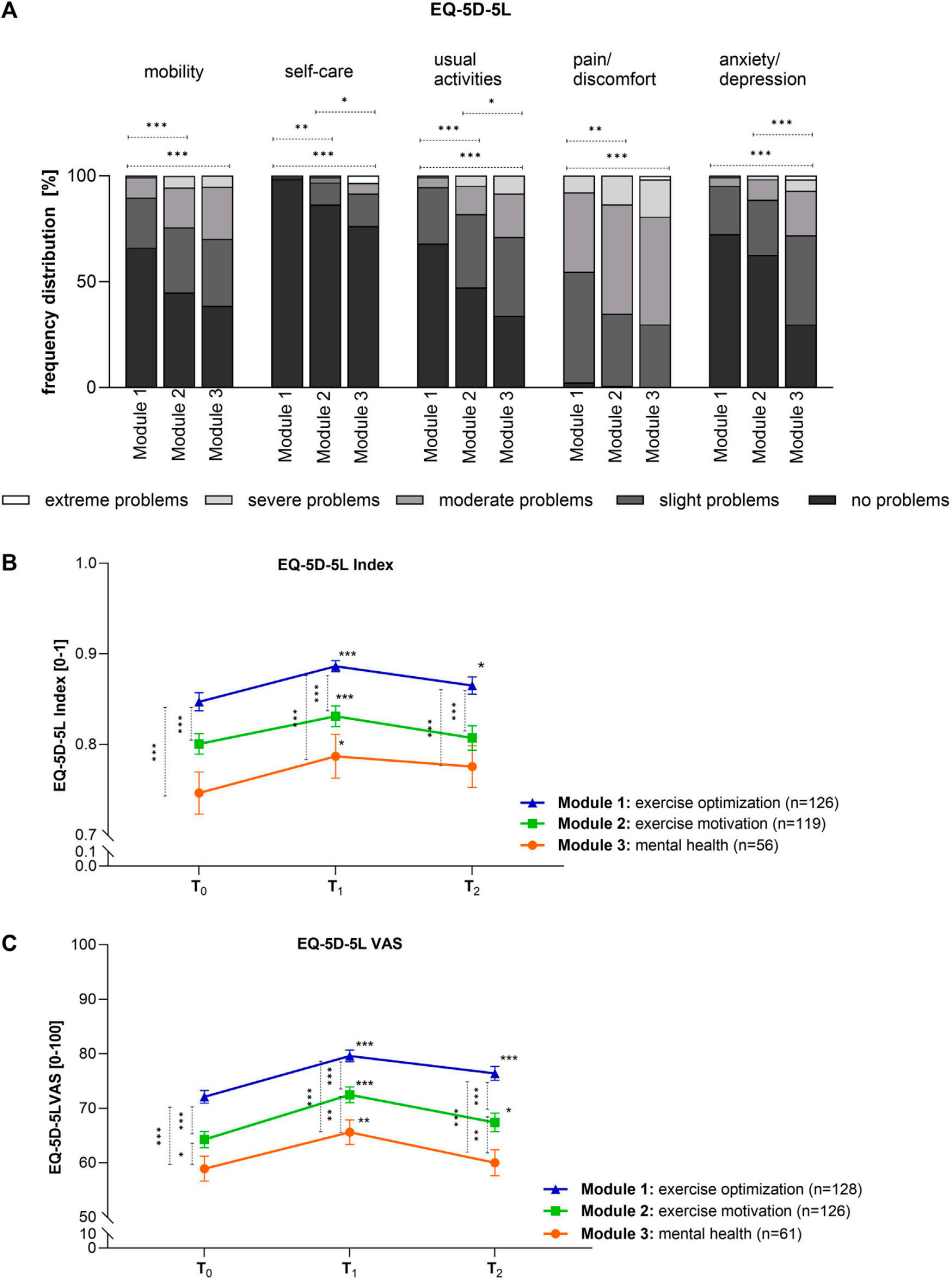
Differences in quality of life: **(A)** The frequency distribution at T_0_ [%] for the five dimensions is shown. Kruskal Wallis test: mobility: ***p < 0.001 (exercise optimization vs. exercise motivation), ***p < 0.001 (exercise optimization vs. mental health); self-care: *p < 0.05 (exercise motivation vs. mental health), **p < 0.01 (exercise optimization vs. exercise motivation), ***p < 0.001 (exercise optimization vs. mental health); usual activities: *p < 0.05 (exercise motivation vs. mental health), ***p < 0.001 (exercise optimization vs. exercise motivation), ***p < 0.001 (exercise optimization vs. mental health); pain: **p < 0.01 (exercise optimization vs. exercise motivation), ***p < 0.001 (exercise optimization vs. mental health); anxiety/depression: ***p < 0.001 (exercise optimization vs. mental health), ***p < 0.001 (exercise motivation vs. mental health). **(B)** The EQ-5D-5L index reflects the positive impact of the GVA measure and the sustained effect to a lesser extent during the measured time points. Values are given as mean ± SEM. Friedmann test: ***p < 0.001 (exercise optimization: T_0_ vs. T_1_), ***p < 0.001 (exercise motivation: T_0_ vs. T_1_), *p < 0.05 (mental health: T_0_ vs. T_1_), *p < 0.05 (exercise optimization: T_0_ vs. T_2_); Kruskal Wallis test: ***p < 0.001 (T_0_ and T_1_: exercise optimization vs. mental health), ***p < 0.001 (T_0_ and T_1_: exercise optimization vs. exercise motivation). **(C)** Values are given as mean ± SEM. Friedmann test: ***p < 0.001 (exercise optimization: T_0_ vs. T_1_; T_0_ vs. T_2_); ***p < 0.001 (exercise motivation T_0_ vs. T_1_); *p < 0.05 (exercise motivation T_0_ vs. T_2_); **p < 0.01 (mental health T_0_ vs. T_1_); Kruskal Wallis test: ***p < 0.001 (all time points: exercise optimization vs. exercise motivation; exercise optimization vs. mental health), *p < 0.05 (T_0_ exercise optimization vs. mental health), **p < 0.01 (T_1_ and T_2_ exercise motivation vs. mental health). (GVA, Austria 2020).

A subgroup analysis of the five dimensions for the different modules was performed at time T_0_ (Figure 4A). More than 66% of the participants in the optimization module, and 45% in the motivation module had no problems in the mobility dimension, however, this value dropped to 39% in the mental health module. The more severe the problem the higher the percentage in this module, compared to the exercise optimization module. Extreme problems were not indicated at all. The same results could be shown for the dimensions of self-care and usual activities. In the area of self-care, an extraordinarily high percentage already showed no problems at the beginning of the GVA program, regardless of the module. It was striking that in the dimension pain/discomfort, almost no patients related “no problems” or “extreme problems.” While more than half of the exercise optimization module reported slight problems, more than 50% of the other two modules showed moderate problems. The frequency distribution of the anxiety/depression dimension clearly showed that substantially more participants in the mental health module had slight to moderate problems (63%) than in the exercise optimization-(27%) and motivation modules (36%). The scores of the individual dimensions result in a 5-digit code representing a health status as an index value, which ranges from 0 (dead) to 1 (complete health), and thus higher values correspond to higher quality of life (Figure 4B). The positive impact of the program on all participants was clearly shown by a significant increase in the EQ-5D-5L Index comparing T_0_ and T_1_. However, this improvement did not last to such an extent till the follow-up after 6 months, but the index value was still better than at the beginning. Differences between the three modules at T_0_ were still visible at time point T_2_. In the second part of the EQ-5D, participants were asked about their current imaginable health status (Figure 4C). Clear significant differences between the modules could be observed at T_0_, with participants in the optimization module reporting the highest value of VAS (72.09). Patients of the exercise-optimization module had the best imaginable health status at all time points. Change due to the health care program was analyzed by comparing the following measurement points with T_0_. The three modules showed significant differences at all examined time points. Directly after the GVA measures, EQ-5D VAS was significantly increased in all three modules by an average of 11%. The individuals in the mental health module, who had a VAS of 65.61, were even temporarily able to reach the values of those in the exercise-motivation module at T_0_, who had a VAS of 64.25. The imaginable health value decreased over time. Nevertheless, the exercise optimization and motivation modules showed, respectively, 6% and 5% better health values than before the health care program.

### Ability of Work

**FIGURE 5:**
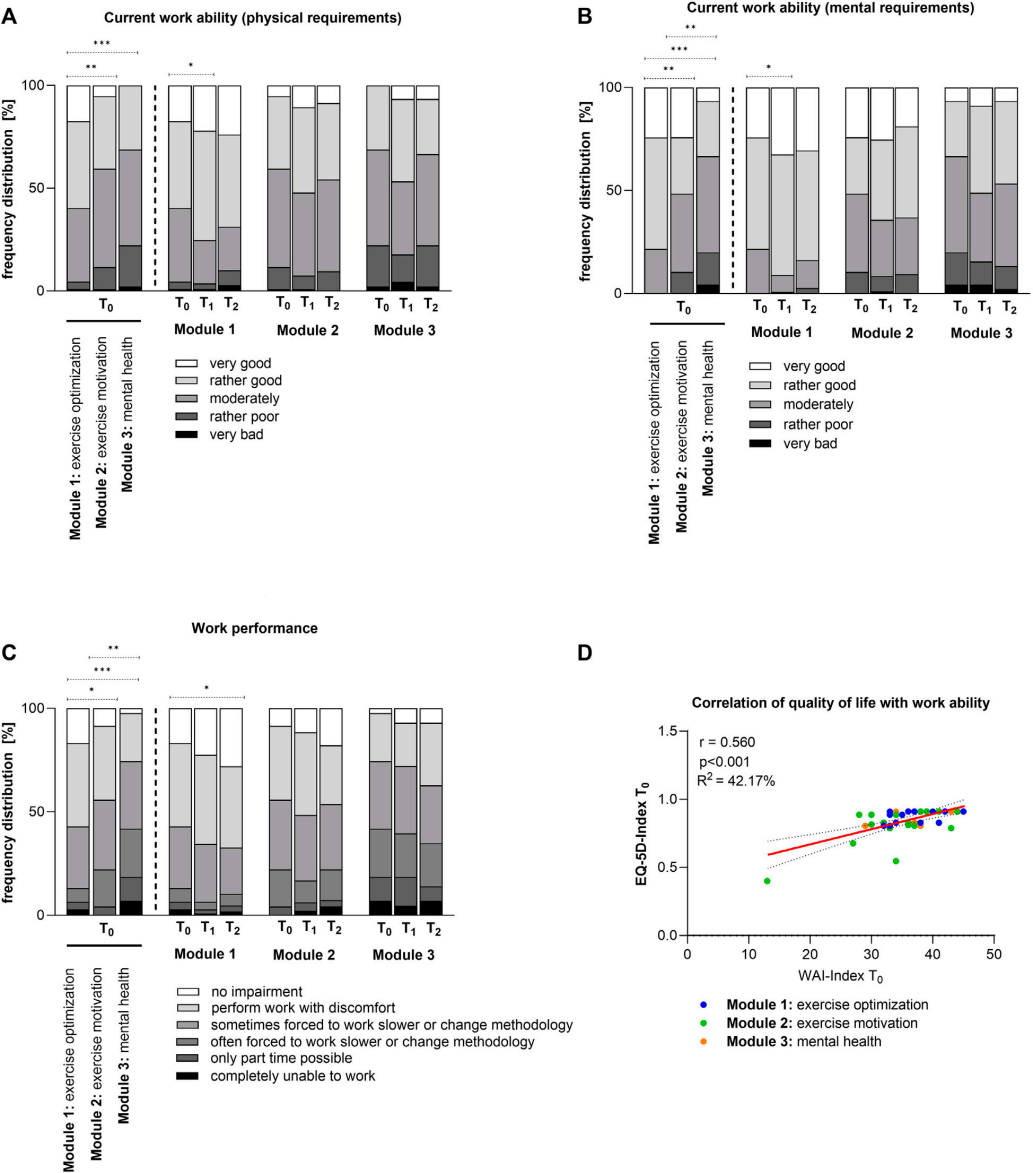
Effect on ability of work: **(A)** Differences in current work ability regarding physical requirements. Kruskal Wallis test: **p < 0.01 (T_0_: exercise optimization vs. exercise motivation), ***p < 0.001 (T_0_: exercise optimization vs. mental health), Friedmann test: *p < 0.05 (exercise optimization: T_0_ vs. T_1_). **(B)** Current work ability regarding mental health requirements for the three modules. Kruskal Wallis test: **p < 0.01 (T_0_: exercise optimization vs. exercise motivation), **p < 0.01 (T_0_: exercise motivation vs. mental health), ***p < 0.001 (T_0_: exercise optimization vs. mental health); Friedmann test: *p < 0.05 (exercise optimization: T_0_ vs. T_1_). **(C)** Impairment of work performance. Kruskal Wallis test: *p < 0.05 (exercise optimization vs. exercise motivation), **p < 0.01 (exercise motivation vs. mental health), ***p < 0.001 (exercise optimization vs. mental health); Friedmann test: *p < 0.05 (exercise optimization: T_0_ vs. T_2_). **(D)** Correlation of quality of life with work ability at time point T_0_. (GVA, Austria 2020).

Work ability analysis was done for employed people (Figure 5). Participants of the exercise optimization module differed significantly in their current work ability regarding physical and mental requirements, by comparing to the two other modules at time point T_0_ (Figure 5A,B; first three bars). Improvements by the program could be observed. People felt better able to cope with the physical requirements over time, thus the proportion with “very good” work ability increased (Figure 5 A). It was striking that in the mental health module, only a few participants rated their work ability with regard to mental demands as very good (Figure 5B). Furthermore, the impairment of work performance was investigated. An initial difference in the subgroups emerged (Figure 5C; first three bars). Approximately 7% of the mental health group estimated their work performance impairment to be so high that they could no longer work at all. More than half reported they were forced to work more slowly. As a result of the measures, more individuals rated their work performance impairment as lower. The extent of improvement depended on the module in which they participated (Figure 5C). The results demonstrated a significant positive correlation (r = 0.560, 95% CI 0.2922–0.7462) between the work ability expressed as WAI-Index and the quality of life (EQ-5D-5L Index) at the beginning of the program (Figure 5D). Nearly 42% of the variability observed in the EQ-5D-5L Index was explained by the ability to work (*R* ^2^ = 42.17%).

## Discussion

Approximately 1.71 billion people worldwide suffer from musculoskeletal conditions, which limit mobility and agility, leading to early retirement, causing subjective distress, reduced well-being, and diminished participation in social life [27–29]. The PVA has launched this new preventive and modularly built health care program “Gesundheitsvorsorge-Aktiv,” which addresses the mental or physical needs of individual participants. The GVA program is intended to prevent more severe impairments in the future and facilitate participation in working life. Patients were assigned to modules by physicians according to their health status. The exercise optimization module (40%) and exercise motivation module (41%) accounted for the largest proportion of participants. However, not to be neglected were also the 19% of all evaluable participants who received psychological support in the mental health module, which indicates that psychological distress often co-occurs with, or increases the risk of developing physical complaints [30]. Sex differences in mental disorders are among the most interesting and substantial findings in the field of psychiatry. For instance, there are differences in prevalence, symptomatology, risk factors, and influencing factors or progression [31, 32]. Women are more likely to show anxiety and depressive symptoms [33]. Consistent with this, the mental health-module was composed of a high proportion of women (66%). Women and men are still exposed to different living conditions and there are various areas with gender-specific characteristics and differences that are biologically or socially determined as well as psychologically shaped. Multiple stresses to which women are exposed repeatedly lead to overstrain syndromes that make psychotherapeutic support necessary [34]. Our data also reflect the statement that two-thirds of psychotherapies are used by women and one-third by men [31, 35]. Poor mental health also affects working life, as reflected in the high percentage of 22% currently not working in the mental health module.

Regular exercise has been proven to be an important building block for positively influencing health and quality of life. One goal of the GVA program is, therefore, to encourage people to enjoy exercise as a basis for a healthy life. It can be clearly seen that the participants of the exercise motivation module and mental health module showed a deficit in activity duration at the beginning. The program provided an impetus to engage in daily activity. The success can be seen in a clear improvement in the sporting duration as well as in frequency. The motivational boost provided by the program was internalized by the participants of the exercise motivation module and an increase in athletic performance was observed after 6 months.

Psychosocial components often carry emotional value and have the potential to cause physical or psychological damage to health [36], They are often associated with stress, which was seen by the initial very high stress level in the mental health module. Numerous studies have demonstrated that they are also associated with musculoskeletal disorders by affecting physical factors or directly through stress symptoms [37]. The significant baseline differences in psychosocial components highlight the importance of offering different modules tailored to the needs of individual patients to maximize the success of interventions. The results suggest that clinicians correctly assigned patients to the modules according to their needs. Patients in the mental health module scored lower in all psychosocial components and therefore needed special interventions based on stress prevention, learning relaxation and distancing mechanisms, and strengthening self-protection mechanisms. Regardless of the initial situation, psychosomatic and depressive symptoms as well as stressors can be successfully reduced by GVA interventions, thus counteracting workplace overload and promoting independence and personal responsibility so that patients can once again play an active role in their family life, at work, and in society. In particular, patients who participated in the mental health module were found to be better able to cope with stress through the learned tools, an effect that can also be shown to have a lasting impact.

In recent years, quality of life has become an important endpoint in medical care and this concept has become common in socio-economic and cultural development [38]. The WHO defines quality of life as an individual’s perception of their life situation regarding their culture, values, and in relation to their goals, expectations, norms, and interests. It is a collective term that usually refers to the degree of subjective well-being of a person and can be influenced by various factors that make up the living conditions of an individual. Material living standards, education, career opportunities, social status, and physical and mental health all play a significant role in this construct [39]. Correlation analyses at T_0_ showed that participants in the modules exercise motivation and mental health had more problems in the individual dimensions than individuals who came for exercise optimization. These deficits were also reflected in lower overall quality of life. In addition, a direct relationship between psychosocial components and the quality of life dimensions was found, showing that offering specific modules is of utmost importance. To improve quality of life, one should work on work-life balance, relationships at work and home, and health. Therefore, the GVA program includes various recommendations and activity courses that are very well received and implemented by the participants, which is also reflected in sustainable improvement. Various studies have shown a positive relationship between physical activity and various indicators of quality of life, mainly in older adults or chronically ill people [40–43], however, no correlation was found in this study.

Active participation in work represents an important point in everyone’s life, as it promotes autonomy, independence, social networks, and an increase in self-esteem. Two important aspects, related to overall well-being, productivity, and employee retention, are job satisfaction and work-related quality of life. Quality of life can be directly linked to work by measuring the non-financial aspects of a job leading to employee satisfaction or dissatisfaction [44–47]. Partially or no longer guaranteed participation in working life may lead to a reduction in quality of life and perhaps even depressive symptoms later on. GVA measures make a significant contribution to improvement and maintenance because a good state of health or more efficient management of any stress factors that may arise can be the basis for better and more effective work performance. This interplay of individual factors is reflected in the different assessments of physical and mental work demands among participants in the three modules at the beginning of the program. The success of the interventions in improving psychosocial components, increasing physical activity, and enhancing quality of life was directly reflected in the assessment of work performance impairment and current work ability regarding psychological, physical, and mental requirements. This again illustrates the importance of biopsychosocial approaches to rehabilitation [48].

### Strengths and Limitations

In this program, the patient can decide for the first time, together and with the support of an expert (physician), which therapy preferences he or she would like to set. The evaluation of the individual modules and their comparison was particularly valuable to find out how well this individual assignment to one of the three modules and its implementation works. The active involvement of the patient in the selection process promotes self-reflection and assessment of his or her own state of health and can increase adherence. The examined results may be particularly useful for holistic approaches to the prevention and rehabilitation of people with musculoskeletal disorders. Aside from these strengths, one limitation is having no control group with no intervention. It is important to keep in mind that individuals who have applied and been approved for the GVA program come to the facility only for these specific programs, and that is one reason why we can evaluate them. The measures are routinely administered, and it is not ethical to deny patients therapeutic uses. Another limiting factor could be the short follow-up of 6 months, however, at the also chosen 12 months follow-up the dropout rate was so high that reliable analyses were not possible. Therefore, in this study, we concentrated on the three time points T_0_ (beginning), T_1_ (after 3 weeks), and T_2_ (6 months after measures). It is known that socioeconomic status has a great impact on health care utilization in general and especially in taking part in preventive programs and that a low status can be a risk factor for many somatic and mental health conditions. However, in this research work, no variables were collected to capture socioeconomic status, which may limit the study. A limiting factor in data collection was the lack of continuous monitoring of completed questionnaires, which resulted in a smaller number of evaluable surveys at the 12 months follow-up, which was the reason why we did not include this time point in this study.

### Conclusion

Looking at the results of the evaluation of this new preventive health care program, it is clear that the modular structure represents a valuable step towards a more personalized approach to health promotion. Thus, more attention is paid to the individual patient’s initial situation in order to apply therapies more effectively. In addition, this program leads to an increase in mobility and an improvement in psychological performance, which in turn provides a valuable basis for increasing work ability. In summary, GVA is a promising program for promoting health and maintaining work ability and represents an enrichment of the methodological spectrum.

## References

[1] Myck M. Living Longer, Working Longer: The Need for a Comprehensive Approach to Labour Market Reform in Response to Demographic Changes. Eur J Ageing (2015) 1:3–5. 10.1007/s10433-014-0332-x PMC4342506 25750608

[2] Bongers PM de Winter CR Kompier MA Hildebrandt VH. Psychosocial Factors at Work and Musculoskeletal Disease. Scand J Work Environ Health (1993) 5:297–312. 10.5271/sjweh.1470 8296178

[3] Klimont J. Hauptergebnisse des Austrian Health Interview Survey (ATHIS) und Methodische Dokumentation; Bundesministerium für Soziales, Gesundheit, Pflege und Konsumentenschutz (BMSGPK). Vienna: Statistics Austria (2019).

[4] Wu A March L Zheng X Huang J Wang X Zhao J Global Low Back Pain Prevalence and Years Lived With Disability From 1990 to 2017: Estimates From the Global Burden of Disease Study 2017. Ann Transl Med (2020) 6:299. 10.21037/atm.2020.02.175 PMC7186678 32355743

[5] Vos T. Global, Regional, and National Incidence, Prevalence, and Years Lived With Disability for 328 Diseases and Injuries for 195 Countries, 1990-2016: A Systematic Analysis for the Global Burden of Disease Study 2016. Lancet (2017) 390:1211–59. 10.1016/S0140-6736(17)32154-2 28919117 PMC5605509

[6] Leoni T. Fehlzeitenreport 2020. Krankheits-und unfallbedingte Fehlzeiten in Österreich: WIFO (2020). Available at: https://EconPapers.repec.org/RePEc:wfo:wstudy:66636 (Accessed December 10, 2020).

[7] U.S. Department of Health and Human Services. Physical Activity Guidelines for Americans. 2nd ed. United States: U.S. Department of Health and Human Services (2018). Available at: https://health.gov/paguidelines/second-edition (Accessed August 24, 2021).

[8] Sjøgaard G Christensen JR Justesen JB Murray M Dalager T Fredslund GH Exercise Is More Than Medicine: The Working Age Population's Well-Being and Productivity. J Sport Health Sci (2016) 2:159–65. 10.1016/j.jshs.2016.04.004 PMC6188718 30356522

[9] European Commission Science Hub. Physical Activity and Sedentary Behaviour (2020). Available at: https://ec.europa.eu/jrc/en/health-knowledge-gateway/promotion-prevention/physical-activity.

[10] Titze S Dorner TE Ropin K Halbwachs C Zeuschner V Stickler T. Warum Österreichische Bewegungsempfehlungen? Gesundheitswesen (2020) 3:S168–169. 10.1055/a-1189-3424 32984941

[11] Verena Z. Austrian Physical Activity Recommendations–Key Messages. Vienna: National Public Health Institute (2020). Available at: https://jasmin.goeg.at/1655/1/fgoe_wb17_bewegungsempfehlungen_e_bf.pdf.

[12] Vargus-Adams JN Majnemer A. International Classification of Functioning, Disability and Health (ICF) as a Framework for Change: Revolutionizing Rehabilitation. J Child Neurol (2014) 8:1030–5. 10.1177/0883073814533595 24850572

[13] Waddell G. Preventing Incapacity in People With Musculoskeletal Disorders. Br Med Bull (2006) 77-78:55–69. 10.1093/bmb/ldl008 16968689

[14] Stucki G Bickenbach J Gutenbrunner C Melvin J. Rehabilitation: The Health Strategy of the 21st century. J Rehabil Med (2018) 4:309–16. 10.2340/16501977-2200 28140419

[15] Stucki G Rubinelli S Bickenbach J. We Need an Operationalisation, Not a Definition of Health. Disabil Rehabil (2020) 3:442–4. 10.1080/09638288.2018.1503730 30325685

[16] Stucki G. Olle Höök Lectureship 2015: The World Health Organization's Paradigm Shift and Implementation of the International Classification of Functioning, Disability and Health in Rehabilitation. J Rehabil Med (2016) 48:486–93. 10.2340/16501977-2109 27240215

[17] Leonardi M Lee H Kostanjsek N Fornari A Raggi A Martinuzzi A 20 Years of ICF-International Classification of Functioning, Disability and Health: Uses and Applications Around the World. Int J Environ Res Public Health (2022) 18:11321. 10.3390/ijerph191811321 PMC9517056 36141593

[18] Pensionsversicherungsanstalt Österreich (PVA). Medizinische Rehabilitation und Gesundheitsvorsorge (2023). Available at: https://www.pv.at/cdscontent/load?contentid=10008.577843.

[19] EuroQol Research Foundation. EQ-5D-5L User Guide. Rotterdam: EuroQol Research Foundation (2019). Available at: https://euroqol.org/publications/user-guides.

[20] Greiner W. Der EQ-5D der EuroQol-Gruppe. In: Schoffski O, editor. Gesundheitsökonomische Evaluationen. Dordrecht: Springer (2012). p. 411–22.

[21] Szende A Janssen B Cabases J. Self-Reported Population Health: An International Perspective Based on EQ-5D. Berlin, Germany: Springer (2014). 29787044

[22] Loewe B Spitzer RL Zipfel S Herzog W. Manual Komplettversion und Kurzform: autorisierte deutsche Version des “Prime MD Patient Health Questionnaire (PHQ)”. 2 Auflage Germany: Universitätsklinikum Heidelberg (2002).

[23] Kroenke K Spitzer RL Williams JB Löwe B. The Patient Health Questionnaire Somatic, Anxiety, and Depressive Symptom Scales: A Systematic Review. Gen Hosp Psychiatry (2010) 4:345–59. 10.1016/j.genhosppsych.2010.03.006 20633738

[24] Amler N Felder S Mau W Merkesdal S Schöffski O des Arbeitsgruppe M. Instrumente zur Messung von Effekten einer Frühintervention auf den Erhalt bzw. die Wiederherstellung der Arbeitsfähigkeit in Deutschland – Stellungnahme einer interdisziplinären Arbeitsgruppe. Gesundheitswesen (2018) 1:79–86. 10.1055/s-0041-110678 26695541

[25] Nordenfelt L. The Concept of Work Ability. Bruxelles, New York: P.I.E. Peter Lang (2008). Available at: https://www.diva-portal.org/smash/record.jsf?pid=diva2:264490.

[26] Ilmarinen J Tuomi K. Past, Present and Future of Work Ability. People Work Res Rep (2004) 65:1–25.

[27] Broadhead WE Blazer DG George LK Tse CK. Depression, Disability Days, and Days Lost From Work in a Prospective Epidemiologic Survey. JAMA (1990) 19:2524–8. 10.1001/jama.1990.03450190056028 2146410

[28] Simon GE VonKorff M Piccinelli M Fullerton C Ormel J. An International Study of the Relation Between Somatic Symptoms and Depression. N Engl J Med (1999) 18:1329–35. 10.1056/NEJM199910283411801 10536124

[29] Sharpe M Peveler R Mayou R. The Psychological Treatment of Patients With Functional Somatic Symptoms: A Practical Guide. J Psychosom Res (1992) 6:515–29. 10.1016/0022-3999(92)90037-3 1640390

[30] Williams A Kamper SJ Wiggers JH O'Brien KM Lee H Wolfenden L Musculoskeletal Conditions May Increase the Risk of Chronic Disease: A Systematic Review and Meta-Analysis of Cohort Studies. BMC Med (2018) 1:167. 10.1186/s12916-018-1151-2 PMC6154805 30249247

[31] Riecher-Rössler A. Fakt oder Artefakt? Geschlechtsspezifische Unterschiede in der Häufigkeit psychischer Störungen. Basel: Neurologie & Psychiatrie (2008). Available at: https://edoc.unibas.ch/22906/1/20160623095700_576b964c073ca.pdf.

[32] Riecher-Rössler A. Prospects for the Classification of Mental Disorders in Women. Eur Psychiatry (2010) 4:189–96. 10.1016/j.eurpsy.2009.03.002 19556110

[33] American Psychological Association. Study Finds Sex Differences in Mental Illness (2018). Available at: https://www.apa.org/news/press/releases/2011/08/mental-illness (Accessed August 18, 2011).

[34] Beiz M Riecher-Rössler A. Geschlechtsspezifische Aspekte in der Psychotherapie (2017). Available at: https://edoc.unibas.ch/65099/1/20180620100512_5b2a0ab885fca.pdf.

[35] Strauß B Hartung J Kächele H. Geschlechterspezifische Inanspruchnahme von Psychotherapie und Soziale Arbeit. Göttingen: Huber (2002). p. 533–47. Available at: https://scholar.google.com/citations?user=jcavxoyaaaaj&hl=de&oi=sra.

[36] Devereux J Rydstedt L Kelly V Weston P Buckle P. The Role of Work Stress and Psychological Factors in the Development of Musculoskeletal Disorders: The Stress and MSD Study, Robens Centre for Health Ergonomics, Guildford, Surrey (2004). HSE Research Report 273. Available at: https://www.hse.gov.uk/Research/rrpdf/rr273.pdf.

[37] Da Silva JMN Dantas DAC da Silva LB de Melo IES de Morais Correia LMA. Assessment of the Influence of Psychosocial Factors on Musculoskeletal Disorder Symptom Intensity. Work (2022) 1:187–200. 10.3233/WOR-205113 34924412

[38] Trzebiatowski J. Quality of Life in the Perspective of Social and Medical Sciences–Classification of Definitions. Hygeia Public Health (2011) 46(1):25–31.

[39] Larson James S. The World Health Organization’s Definition of Health: Social Versus Spiritual Health. Soc Indicators Res (1996) 38(2):181–92. 10.1007/bf00300458

[40] Elavsky S McAuley E Motl RW Konopack JF Marquez DX Hu L Physical Activity Enhances Long-Term Quality of Life in Older Adults: Efficacy, Esteem, and Affective Influences. Ann Behav Med (2005) 2:138–45. 10.1207/s15324796abm3002_6 16173910

[41] Rejeski WJ Mihalko SL. Physical Activity and Quality of Life in Older Adults. J Gerontol A Biol Sci Med Sci (2001) 56(2):23–35. 10.1093/gerona/56.suppl_2.23 11730235

[42] Courneya KS Friedenreich CM. Physical Exercise and Quality of Life Following Cancer Diagnosis: A Literature Review. Ann Behav Med (1999) 2:171–9. 10.1007/BF02908298 10499138

[43] Vagetti GC Barbosa Filho VC Moreira NB de Oliveira V Mazzardo O de Campos W. Association Between Physical Activity and Quality of Life in the Elderly: A Systematic Review, 2000-2012. Braz J Psychiatry (2014) 1:76–88. 10.1590/1516-4446-2012-0895 24554274

[44] Chen A-H Jaafar SN Noor ARM. Comparison of Job Satisfaction Among Eight Health Care Professions in Private (Non-Government) Settings. Malays J Med Sci (2012) 2:19–26. PMC3431743 22973134

[45] Zubair MH Hussain LR Williams KN Grannan KJ. Work-Related Quality of Life of US General Surgery Residents: Is It Really So Bad? J Surg Educ (2017) 6:e138–46. 10.1016/j.jsurg.2017.09.018 28988955

[46] Mount M Ilies R Johnson E. Relationship of Personality Traits and Counterproductive Work Behaviors: The Mediating Effects of Job Satisfaction. Personnel Psychol (2006) 3:591–622. 10.1111/j.1744-6570.2006.00048.x

[47] Wegge J Schmidt K-H Parkes C Dick R. Taking a Sickie: Job Satisfaction and Job Involvement as Interactive Predictors of Absenteeism in a Public Organization. J Occup Organizational Psychol (2007) 1:77–89. 10.1348/096317906x99371

[48] Engel GL. The Need for a New Medical Model: A Challenge for Biomedicine. Science (1977) 4286:129–36. 10.1126/science.847460 847460

